# Polypyrrole functionalized MoS_2_ for sensitive and simultaneous determination of heavy metal ions in water†

**DOI:** 10.1039/d4ra05688d

**Published:** 2025-01-03

**Authors:** K. S. Manjunatha Kumara, Aisha Siddiqa, P. Shiva Kumar, Golla Lavanya, Srinivasa Budagumpi, Gurumurthy Hegde, D. H. Nagaraju, N. Usha Rani

**Affiliations:** a Department of Chemistry, School of Applied Sciences, REVA University Bangalore 560064 Karnataka India dhnagu@gmail.com; b Center for Nano and Material Sciences, JAIN (Deemed-to-be-University) Jakkasandra Ramanagar (D) 562112 Karnataka India; c Centre for Advanced Research and Development (CARD), CHRIST University Hosur Road Bangalore 560029 Karnataka India; d Department of Freshman Engineering, PVP Siddhartha Institute of Technology Vijayawada 520007 Andhra Pradesh India

## Abstract

Assessing heavy metal ion (HMI) contamination to sustain drinking water hygiene is a challenge. Conventional approaches are appealing for the detection of HMIs but electrochemical approaches can resolve the limitations of these approaches, such as tedious sample preparation, high cost, time consuming and the need for trained professionals. Here, an electrochemical approach is developed using a nano-sphered polypyrrole (PPy) functionalized with MoS_2_ (PPy/MoS_2_) by square wave anodic stripping voltammetry for the detection of HMIs. The developed sensor can detect Pb^2+^ with a limit of detection of 0.03 nM and a sensitivity of 36.42 μA nM^−1^. Additionally, the PPy/MoS_2_ sensor was employed for the simultaneous detection of HMIs of Cd^2+^, Pb^2+^, Cu^2+^ and Hg^2+^. The reproducibility, stability and anti-interference studies confirm that the sensor can be used to monitor HMI contamination of water.

## Abstract 2

Assessing heavy metal ion (HMI) contamination to sustain drinking water hygiene is a challenge.

## Graphical abstract

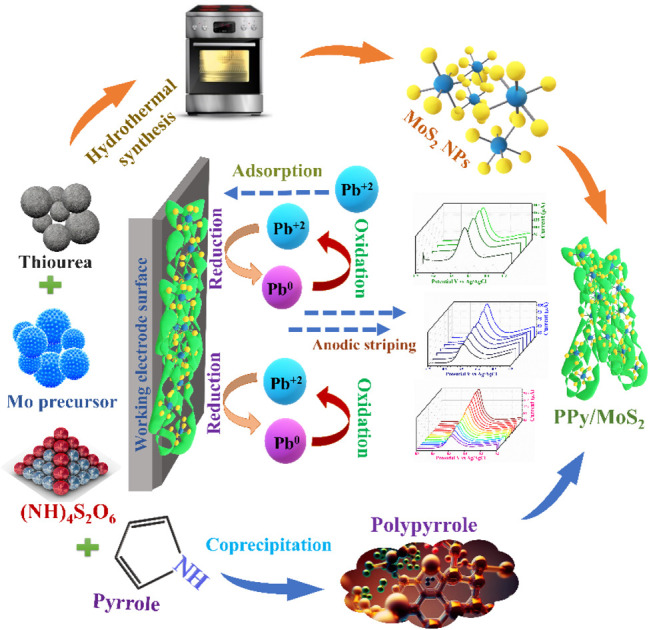

## 1. Introduction

The accessibility of essential, consumable water for life is limited, despite the abundance of water. Globally, 1.1 billion people consume contaminated and unhygienic drinking water, which causes various afflictions. Hence, it is imperative to monitor the quality of water. Emerging industries typically release more environmentally hazardous pollutants, particularly heavy metal ions (HMIs).^1^ HMIs are ubiquitous and their hazardous characteristics, such as chemical stability, non-degradability, bio-accumulation and adverse effects on the environment and human health, have piqued public interest.^2^ Lead ions (Pb^2+^) are a prevalent HMI pollutant with high toxicity even at very low concentrations. Pb^2+^ contamination leads to serious effects on human health.^3^ For instance, Pb^2+^ accumulation in young children may lead to long-term brain and nervous system damage, whereas in adults exposure can result in long-term consequences such as hypertension and renal damage. Prolonged exposure to such pollutants may culminate in carcinogenic illness.^4^ Consequently, HMI contamination must be detected and nullified in drinking water before it is supplied. Worldwide, pollution caused by the HMI is regulated by national and international water authorities.^5,6^ HMI detection is still a major challenge at the trace level, with complex sampling procedures, and hence there is a need to develop sophisticated, simple, inexpensive as well as sensitive devices.

Conventional methods have drawbacks such as tedious sample preparation, pre-concentration steps, the involvement of specialists for instrument operation and complex evaluation procedures.^7^ This can be overcome using electrochemical methods, such as potentiometry, amperometry and impedance techniques, which are promising for detecting HMIs as these are simple to operate in devices and less expensive.^8^ Square wave anodic stripping voltammetry (SWASV) is one of the effective potentiometric methods used to detect HMIs because it offers high sensitivity, high precision, broad measuring range, ease of use and is suitable for field applications.^9^ SWASV mainly depends on the nature of the catalyst, hence the catalyst surface and components have a prevailing role in SWASV detection of HMIs.

Over the last decades, nanomaterials based on metal oxides,^10^ metal sulfides,^11^ layered double hydroxides,^12^ metal/carbonaceous^13^ and organo–inorganic composites^14^ combined with conducting polymers (CPs), such as polyaniline (PANI),^15,16^ polypyrrole (PPy) and poly(3,4-ethylene-dioxy-thiophene) (PEDOT),^17^ have been developed as electrode materials for the detection of HMIs. Molybdenum disulfide (MoS_2_) is one of the most widely used electrode materials in energy conversion,^18,19^ electrochemical sensors and photocatalysis applications, due to its remarkable stability and the different nanostructures that can be synthesized. For instance, Dharmender Singh Rana *et al.* synthesized a 2D nanocomposite of MoS_2_/rGO by a single-step hydrothermal method for the electrochemical detection of mercury (Hg). The sensor exhibited an excellent limit of detection (LOD) of 1.6 μM. This low LOD may be attributed to the availability of sulfur atoms as active sites to increase the sensitivity of the catalyst.^20^ Xi Chen *et al.* prepared rGO/MoS_2_ composite for Hg^2+^ detection by an ultrasonic method with MoS_2_ and rGO by thermal annealing treatment. The rGO/MoS_2_ sensor displayed a low LOD of 1 nM.^21^ Further, by utilizing the synergistic interaction of reduced graphene oxide, molybdenum disulfide and chitosan (rGO/MoS_2_/Cs), Chuanen Guo *et al.* designed a sensitive sensor for Pb^2+^ detection.^22^ SWASV was employed to study the stripping behavior of Pb^2+^. Arya Nair J. S. *et al.* fabricated an electrochemical sensor of partially reduced graphene oxide-nano molybdenum disulfide-modified glassy carbon electrode (GCE) (prGO-MoS_2_/GCE) for the simultaneous detection of Pb^2+^ and Cd^2+^ in drinking water. The study revealed an LOD for Pb^2+^ and Cd^2+^ of approximately 0.0002 ppt and 0.1 ppt, respectively, highlighting the efficacy of the prGO-MoS_2_/GCE in complying with regulatory standards.^23^ Saisree S. *et al.* investigated an electrochemical sensor utilizing sulfur co-doped nitrogen-graphene quantum dots (S,N-GQD) synthesized from polyaniline, with H_2_SO_4_ serving as both the acid catalyst and S-doping agent, through a straightforward hydrothermal synthesis method. The S,N-GQDs demonstrated simultaneous sensing capabilities for three toxic metal ions: Cd^2+^, Pb^2+^ and Hg^2+^. The detection limits achieved for Cd^2+^, Pb^2+^ and Hg^2+^ were notably low, at 1 pM, 10 pM and 1 pM, respectively, marking a significant advancement in the simultaneous detection of these metal ions. The sensitivity values obtained from the respective linear dynamic ranges were 12, 13 and 5 μA μM^−1^ cm^−2^. The LOD values for Cd^2+^, Pb^2+^ and Hg^2+^ were found to be 10^−12^, 10^−11^ and 10^−12^ M, respectively.^24^

As mentioned, nanomaterials and CP composites are promising for the detection and removal of HMIs. Functionalization of the PPy matrix with MoS_2_: (a) increases the surface area to volume ratio; (b) provides a high number of catalytic sites and strong adsorption ability; (c) the sulfur atoms on the surrounding MoS_2_ act as soft a Lewis base with a high affinity towards soft metal ions; and (d) the MoS_2_ may reduce the agglomeration of PPy. In this regard, the functionalization of conducting PPy with MoS_2_ is a way to avoid the limitations for constructing electrochemical sensors.

Herein, we have developed a nano-sphered PPy-functionalized MoS_2_ electrode material to achieve highly sensitive detection of Pb^2+^. MoS_2_ was synthesized by a simple hydrothermal process and PPy/MoS_2_ was synthesized by oxidative polymerization of pyrrole monomer in the presence of MoS_2_. The PPy/MoS_2_ combination provided a platform for the electrochemical detection of HMIs, attributable to the synergistic effect of the PPy and MoS_2_ towards Pb^2+^. The PPy provides a high surface area and the free sulfur groups of MoS_2_ provide active sites of adsorption in the PPy/MoS_2_ sensor, and necessary conductivity. Through the synergistic effect, the PPy/MoS_2_ exhibited a good electrochemical response towards Pb^2+^ by SWASV, with an LOD of 0.03 nM and a sensitivity of 36.42 μA nM^−1^. The primary advantage of our composite material lies in the surface modification of MoS_2_ with the PPy. This modification enhances the number of functional groups available for chelating HMIs through electrostatic interactions. Additionally, polymers are known for their excellent responsiveness to stimuli and provide the requisite conductivity for effective electrochemical sensing. This combination significantly enhances the sensitivity of the sensor.

## 2. Materials and methods

### 2.1 Materials

Ammonium hepta-molybdate ((NH_4_)_6_Mo_7_O_24_·4H_2_O) was obtained from Thermo Fisher Scientific India Pvt. Ltd. Thiourea (CH_4_N_2_S) was purchased from S D Fine-Chem Ltd (SDFCL). Ammonium persulphate ((NH)_4_S_2_O_6_) was obtained from Isochem Laboratories. Pyrrole (99%) was purchased from Sigma-Aldrich. Perchloric acid was procured from Thermo Fisher Scientific India Pvt. Ltd. All chemicals were utilized without any further purification and the solutions were prepared with distilled water.

### 2.2 Synthesis of MoS_2_, and PPy/MoS_2_

MoS_2_ NPs were synthesized through a simple hydrothermal process.^25^ Briefly, a known amount of ammonium hepta-molybdate and thiourea (1 : 1 M/M) was dissolved in 40 mL distilled water. The reaction mixture was transferred to a Teflon-lined autoclave and heated at 180 °C for about 24 hours.^25,26^ The reaction in the autoclave resulted in a black-coloured precipitate. The precipitate obtained was centrifuged, washed with distilled water and ethanol (1 : 1) and allowed to dry overnight at 80 °C.

**Fig. 1:**
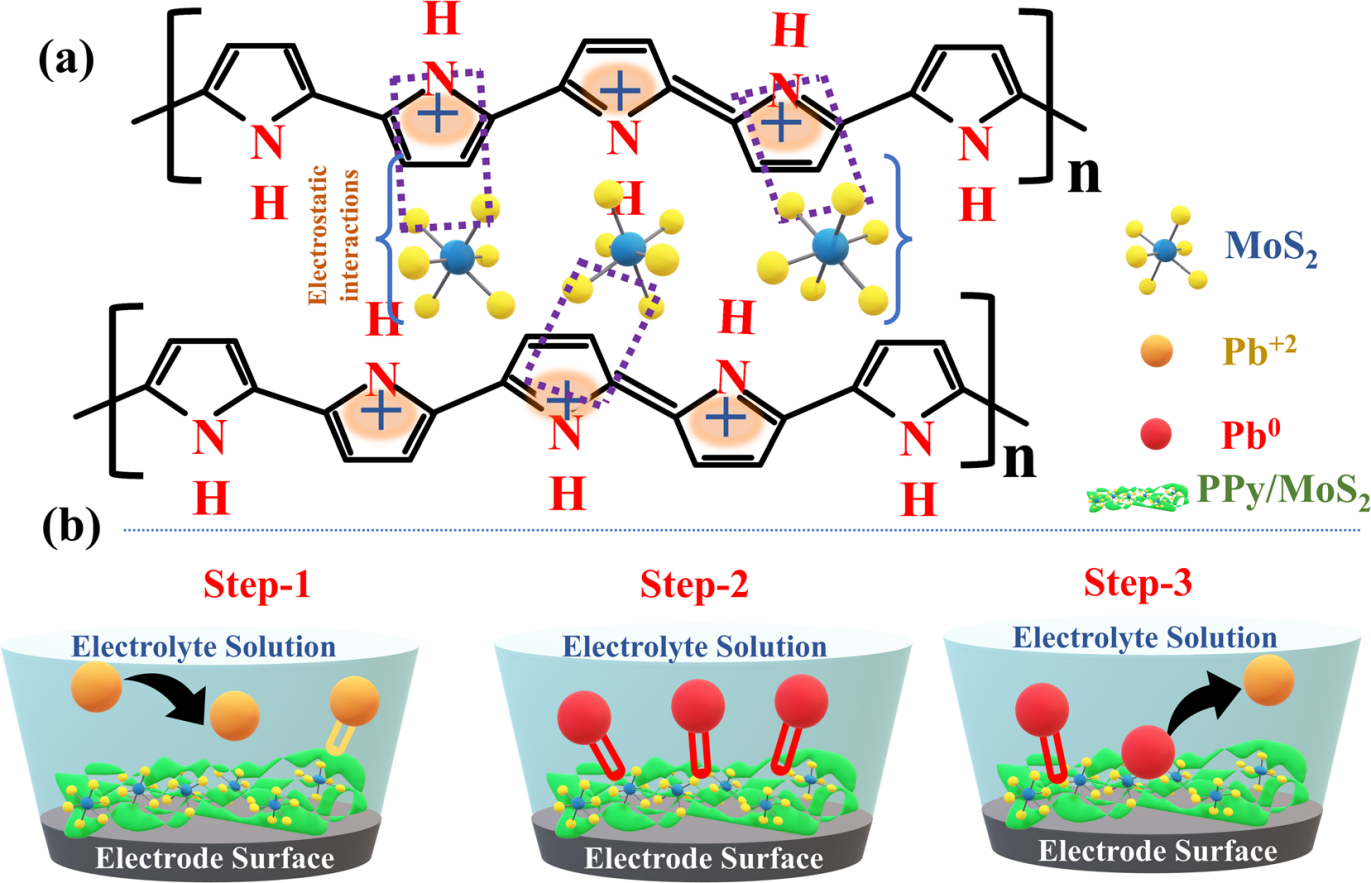
(a) Possible functionalization mechanism of MoS_2_ into the PPy polymer skeleton. (b) Schematic illustration of SWASV for detection of Pb^2+^ by PPy/MoS_2_ sensor.

PPy and PPy/MoS_2_ were synthesized by the oxidative polymerization method. In brief, 10 μL pyrrole was dissolved in 20 mL 0.1 M HClO_4_ and kept in an ice bath (below 5 °C) for about 1 hour. To the reaction mixture, 20 mL of 0.1 M (NH_4_)_2_S_2_O_8_ in 0.1 M HClO_4_ was added dropwise with constant stirring in the ice bath for about 1 hour. After completion of the polymerization, a dark green-coloured precipitate was obtained, washed with distilled water several times and dried overnight at 60 °C.^27^ Similarly, PPy/MoS_2_ was synthesized by oxidative polymerization, by mixing 10 mL pre-dispersed MoS_2_ and 10 μL pyrrole dissolved in 10 mL of 0.1 M HClO_4_ in 1 : 2, 1 : 1 and 2 : 1 ratios (w/w), designated as PPy/MoS_2_-1, PPy/MoS_2_-2 and PPy/MoS_2_-3, respectively, and kept in an ice bath (below 5 °C) for about 1 hour. To this mixture, 0.1 M (NH_4_)_2_S_2_O_8_ in 0.1 M HClO_4_ was added dropwise with constant stirring in the ice bath for about 1 hour.^28^ After completion of the reaction, a cleaning and drying process were followed. The possible mechanism for the functionalization of PPy with MoS_2_ is illustrated in Fig. 1a.

### 2.3 Measurements and electrode preparation

Following the synthesis, electrochemical measurements (SWASV) were performed with a traditional three-electrode system, in which Ag/AgCl was used as the reference electrode, a modified GCE as the working electrode and Pt wire as the counter electrode. SWASV was performed in 0.1 M sodium acetate (Na–Ac) buffer under optimized parameters at a scan rate of 100 mV s^−1^. Before the electrochemical measurements, the GCE was pre-polished using alumina slurry of different particle sizes on appropriate padding, followed by electrochemical cleaning by cyclic voltammetry in 0.5 M H_2_SO_4_. A 10 μL portion of 1 mg of PPy/MoS_2_ dispersed in 1 mL water : ethanol (1 : 1) solution was drop-cast onto the clean GCE. Structural confirmation of the PPy/MoS_2_ sensor was obtained by characterization by X-ray diffraction (XRD) with a Rigaku SmartLab instrument scanning at 3° min^−1^ in the range 5–80°, and by Raman spectroscopy using an Olympus BX microscope and HORIBA Scientific CCD detector. The surface morphology was analysed by field-emission scanning electron microscopy (FESEM) with a Tescan-Mira 3 LMH model, and FESEM equipped with energy dispersive X-ray (EDAX) and elemental analysis was obtained using a QUANTAX 200 with XFlash BRUKER.

### 2.4 Mechanism of functionalization of MoS_2_ into PPy and HMI detection by PPy/MoS_2_ sensor

Functionalization is a process of oxidizing or reducing the polymer (PPy) simultaneously and introducing counter anions or cations, respectively. Depending on the nature of the materials, three significant interactions influence the functionalization process: electrostatic interactions, hydrogen bonding and π–π interactions.^29^ The conductivity of PPy arises from electron transfer along the conjugated polymer, as well as the motion of charge carriers. Oxidation of PPy results in a positive net charge over the surface of the PPy, resulting in the bipolaron form of PPy, which can be counterbalanced by the anions and become a part of the PPy backbone. The anions might be released back during reduction of the polymer.^30,31^ With this scenario, MoS_2_ was functionalized into the PPy skeleton aided by electrostatic interactions between the positive charge on PPy and the negative sulfur charge of MoS_2_, as shown in Fig. 1a. Considering MoS_2_ is bulky by nature, release back into the electrolyte by reduction is not desirable.^32^ Sensitivity towards HMIs can be achieved by electro-hopping in PPy/MoS_2_. The conductivity of PPy is driven by conjugation, with functionalization of PPy developing electron-rich centres that can be used as electro-hopping sites in the PPy skeleton, potentially increasing the conductivity.

PPy, PANI and PEDOT are well-recognized for the availability of numerous N–H functional groups, whereas^33^ MoS_2_ is recognized for its readily available free sulfur groups. The combined accessibility of the functional groups on individual PPy and MoS_2_ of the PPy/MoS_2_ positively influences the detection of Pb^2+^. Conversely, sulfur and amine groups, being negatively charged, tend to attract positively charged Pb^2+^ effectively onto the electrode surface by electrostatic interaction. During electrochemical detection of Pb^2+^, the SWASV mechanism typically consists of three basic steps of pre-concentration and stripping of HMIs, as illustrated in Fig. 1b. The first step involves the amine and sulfur groups of the catalyst to form a metal complex on the surface of the working electrode. In step 2, Pb^2+^ is reduced to Pb^0^ during pre-concentration under the influence of the deposition potential. Finally, in step 3, anodic stripping of Pb occurs, where Pb^0^ is oxidized to Pb^2+^, releasing the ions back into the electrolyte solution, which results in a stripping voltammogram, where the stripping peak current is directly proportional to the concentration of Pb^2+^ in the electrolyte.^34^ In the case of the detection of Pb^2+^ using MoS_2_, the process benefits from both a strong adsorption affinity and an electrochemical (EC) reduction process. The sulfur (S^−2^) in MoS_2_ acts as an electron donor, facilitating the reduction of Pb^2+^ to Pb^0^. Consequently, the removal of Pb^2+^ is largely driven by the affinity of MoS_2_ for the metal ion rather than solely by a strong EC mechanism.

Further enhancing the performance, the oxidative polymerization of pyrrole to form PPy functionalizes the surface of MoS_2_. This functionalization increases the number of nitrogen- and oxygen-containing functional groups, improving interactions with HMIs. In addition, PPy provides a higher surface area, which also facilitates better mass transfer of the analyte to the electrode surface and reduces the thickness of the diffusion layer. This ensures that more metal ions can reach the electrode quickly, resulting in a faster electrochemical process.

## 3. Results and discussion

### 3.1 Powder XRD and Raman spectroscopy

**Fig. 2:**
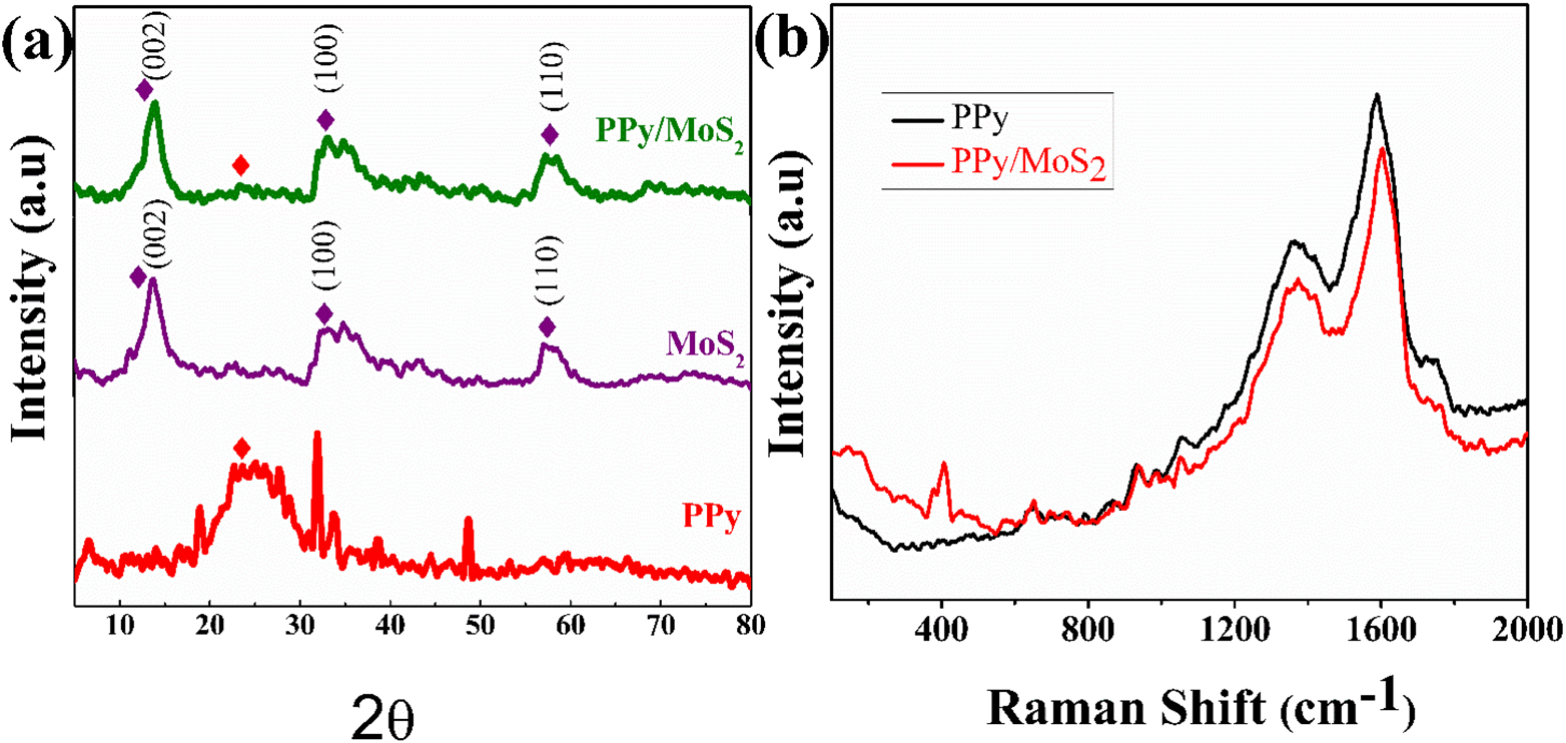
(a) XRD of PPy, MoS_2_ and PPy/MoS_2_. (b) Raman spectra of PPy and PPy/MoS_2_.

The structure and purity of PPy, MoS_2_ and functionalized PPy/MoS_2_ were investigated through XRD, as demonstrated in Fig. 2a. The XRD pattern of bare PPy exhibits a weak and broad peak at 21.5°, which is correlated to the short-range arrangement of the chains and amorphous nature of PPy.^35^ The diffraction peaks at 2*θ* values of 13.79°, 33.00° and 57.19° can be indexed as the 002, 100 and 110 crystal diffraction of MoS_2_ (JCPDS No. 37-1492).^36,37^ Meanwhile, the broad diffraction peak at 2*θ* = 21.5° corresponds to the PPy of PPy/MoS_2_. The diffraction peaks offer favourable evidence for the functionalization of PPy with MoS_2_. Furthermore, the Raman spectra were used to examine the characteristics of PPy and PPy/MoS_2_, as illustrated in Fig. 2b. The C=C backbone stretching and ring stretching modes are indicated by the two broad peaks at 1354.91 and 1595.18 cm^−1^, respectively, in the PPy Raman spectrum.^38^ The PPy/MoS_2_ spectrum shows two characteristic peaks at 376.83 and 409.91 cm^−1^, which correspond to the in-plane E_2g_ and out-of-plane A_1g_ vibration modes of MoS_2_, respectively.^39^ The appearance of the C=C backbone stretching and the ring stretching modes of PPy confirmed the successful functionalization of MoS_2_ into the PPy backbone.

### 3.2 FESEM, EDAX and elemental mapping

**Fig. 3:**
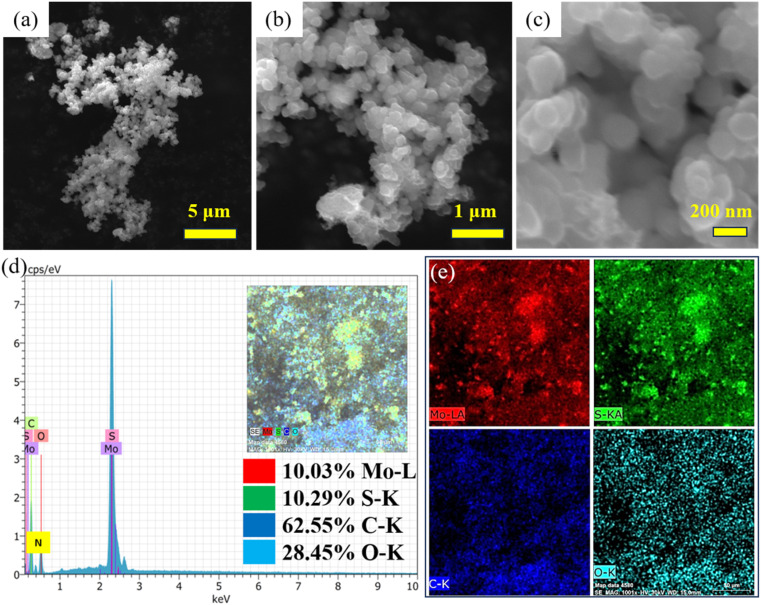
(a–c) Low- to high-magnification FESEM images of PPy/MoS_2_. (d and e) EDAX and elemental mapping of PPy/MoS_2_.

Fig. 3a–c shows low- to high-magnification FESEM images of the PPy/MoS_2_ sample. The morphology obtained was found to be irregular-sized nanospheres. Moreover, the MoS_2_ nanospheres were agglomerated in combination with PPy. The polymer matrix plays an important role in inducing the spherical morphology of MoS_2_, by interacting with it.^40,41^ FESEM equipped with EDAX confirmed the presence of the elements Mo, S, C, O and N in the PPy/MoS_2_, with elemental weight ratios as indicated in Fig. 3d. The elemental mapping verified the uniform distribution of the elements in the material, as shown in Fig. 3e.

### 3.3 Electrochemical detection of HMIs

**Fig. 4:**
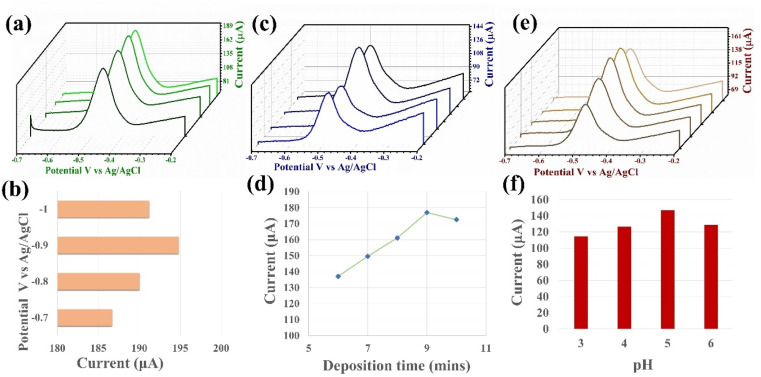
(a) Optimization of deposition potential on SWASV detection of Pb^2+^ by PPy/MoS_2_/GCE (pH = 5.0 (0.1 M Na–Ac), deposition time (min) = 10 and [M]^2+^ = 200 nM) and (b) respective bar graph of optimization of deposition potential from −0.7 to −1.0. (c) Optimization of deposition time on SWASV detection of Pb^2+^ by PPy/MoS_2_/GCE (pH = 5.0 (0.1 M Na–Ac), deposition potential (V) = −0.9 and [M]^2+^ = 200 nM) and (d) respective line graph of optimization time from 6 to 10 min. (e) Optimization of pH (0.1 M Na–Ac) for SWASV sensitivity of PPy/MoS_2_/GCE (deposition potential (V) = −0.9, deposition time (min) = 10 and [M]^2+^ = 200 nM) and (f) respective bar graph of optimization of pH from 3 to 6.

The SWASV response is sensitive to the deposition potential, deposition time and pH of the electrolyte. Therefore, we investigated the optimal experimental conditions for the detection capability of the PPy/MoS_2_ sensor. The effective deposition potential, time and pH for the electrolyte of Pb^2+^ was determined by changing each of these parameters in turn while detecting Pb^2+^ under the same conditions.^42^ As shown in Fig. 4(a and b), as the deposition potential increases the peak current also increases, with a maximum peak current at −0.9 V *vs.* Ag/AgCl, and then decreases with further increase in the potential. When the potential exceeds −0.9 V, the peak current decreases mainly due to the hydrogen evolution reaction by water splitting. H_2_ bubbles at the electrode surface hinder the active site, which is initially available for Pb^2+^ adsorption, resulting in the reduction of the peak current.^8,43^ Hence, −0.9 V *vs.* Ag/AgCl was employed as the optimal deposition potential for the further detection processes. The effect of deposition time on the peak current responses for Pb^2+^ is illustrated in Fig. 4(c and d). The maximum peak current was achieved with a deposition time of 9 min. With further increase in the deposition time, the peak current remains almost constant, which could be due to saturation of the active sites on the sensor surface. Hence, 9 min of deposition time was employed as the optimal deposition time for further detection.

The pH of the buffer (Na–Ac) solution has been shown to have a significant impact on the sensitivity of SWASV for targeted metal ions. Therefore, the influence of the pH of the Na–Ac buffer on stripping of Pb^2+^ was examined, as illustrated in Fig. 4(e and f). As the pH increased in the range from 3 to 6, the peak current increased, up to a pH of 5. When the pH was greater than 5.0 (basic), the peak current decreased, which may be due to possible reduction of H^+^ to H_2_ occurring at the electrode surface. H^+^ reduction may influence the reduction of Pb^2+^ ions.^44^ Hence, pH 5 was employed as the optimized pH for the Na–Ac buffer for further detection of Pb^2+^.

**Fig. 5:**
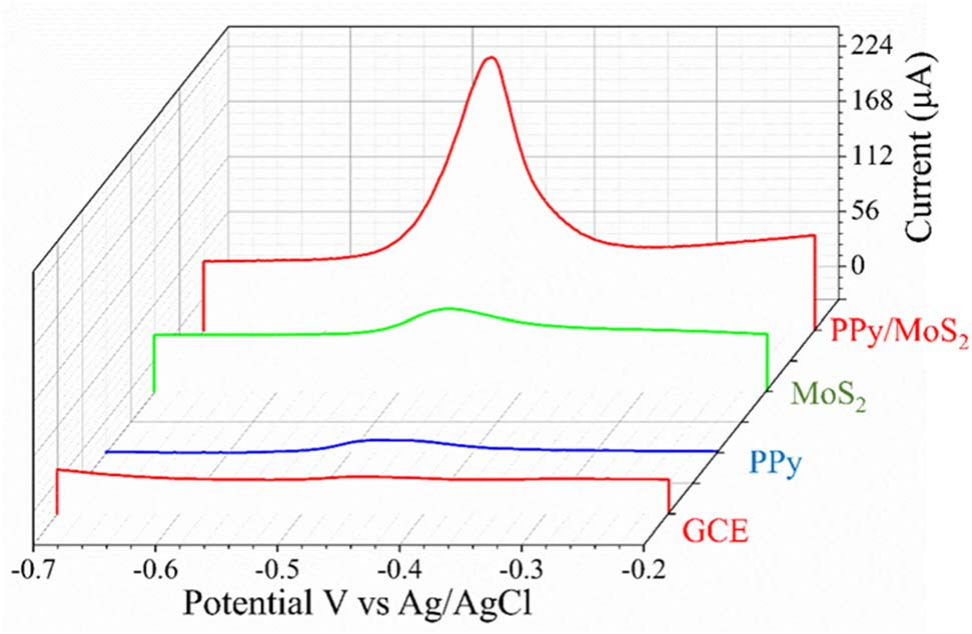
SWASV comparison of GCE, PPy, MoS_2_ and PPy/MoS_2_ electrodes for the detection of 300 nM Pb^2+^ concentration.

To evaluate the square wave response of modified electrodes to Pb^2+^, experiments were conducted on electrodes modified with different materials by the SWASV method. SWASV sensing behavior of Pb^2+^ at bare GCE, PPy, MoS_2_ and PPy/MoS_2_ electrodes for the detection of 300 nM Pb^2+^ concentration in a potential range −1.0 to 0.0 V *vs.* Ag/AgCl is shown Fig. 5. The peak current generated by PPy/MoS_2_/GCE significantly improved, indicating that PPy had a good effect on improving the detection of Pb^2+^. In particular, with the synergistic effect with MoS_2_, the peak current signal increased significantly compared to that of bare GCE, PPy and MoS_2_/GCE. This was mainly because PPy had abundant functional groups to facilitate enrichment of Pb^2+^, thereby further improving the sensitivity of the Pb^2+^ detection.

**Fig. 6:**
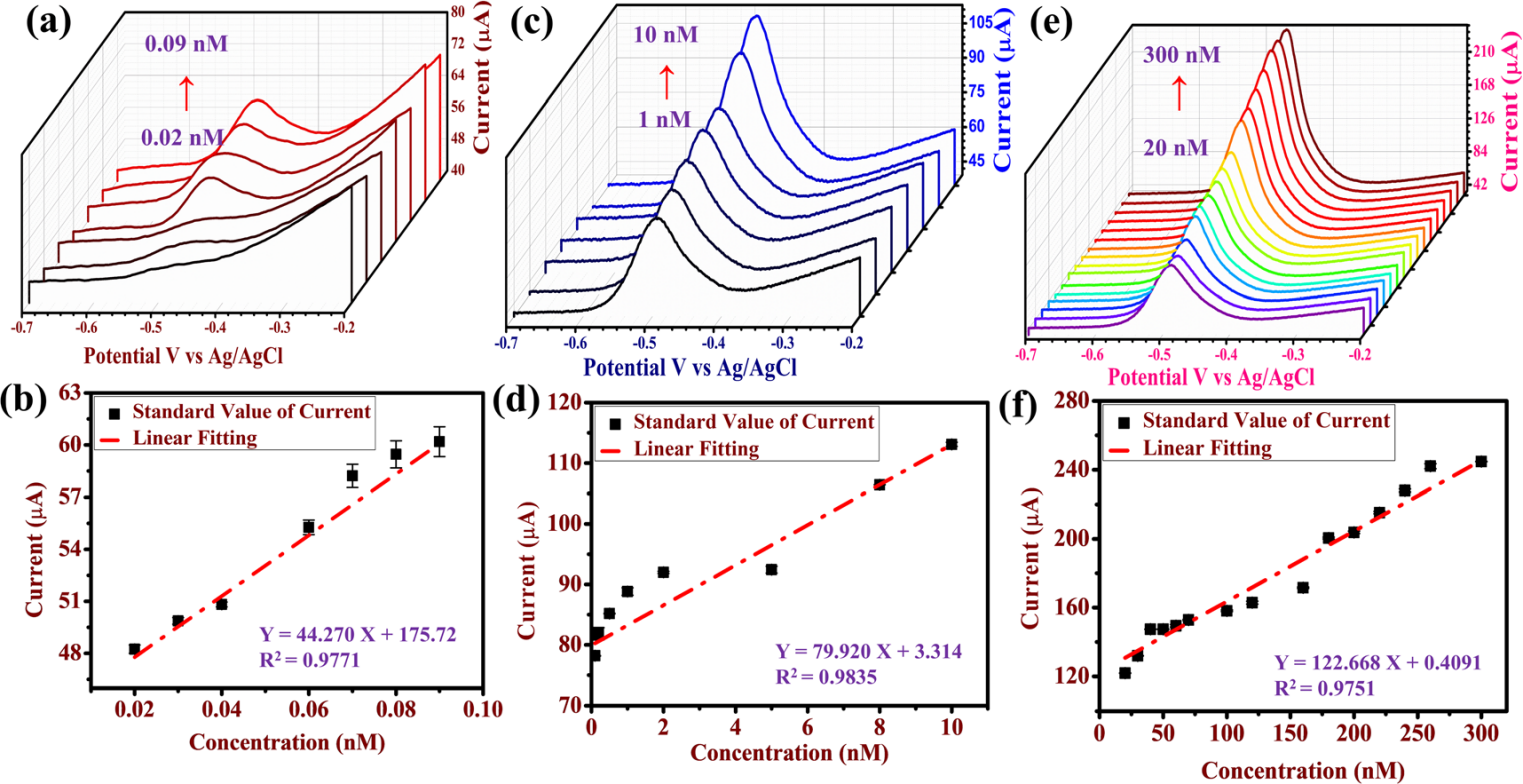
(a) SWASV detection of Pb^2+^ with a range of 0.02 to 0.09 nM and (b) its respective calibration plot. (c) SWASV detection of Pb^2+^ with a range of 1 to 10 nM and (d) its respective calibration plot. (e) SWASV detection of Pb^2+^ with a range of 20 to 300 nM and (f) its respective calibration plot, under optimal SWASV experimental conditions.

Individual ion detection with various concentrations of Pb^2+^ was carried out under optimal conditions. Fig. 6 illustrates the SWASV response of Pb^2+^ concentrations from 0.02 to 300 nM, which demonstrates that as the concentration increases the peak current increases. The linear fit between the peak current and concentration of Pb^2+^ is shown in Fig. 6, with a linear equation of *Y* = 44.270*X* + 175.72 (*R*^2^ = 0.9771) at concentrations from 0.02 to 0.09 nM of Pb^2+^ (Fig. 6a and b), linear equation of *Y* = 79.920*X* + 3.314 (*R*^2^ = 0.9835) at concentrations from 0.1 to 10 nM of Pb^2+^ (Fig. 6c and d) and linear equation of *Y* = 122.668*X* + 0.4091 (*R*^2^ = 0.9751) at concentrations from 20 to 300 nM of Pb^2+^ (Fig. 6e and f). *Y* corresponds to the peak current and *X* is the concentration. LOD (S/N = 3) was calculated to be 0.033 nM (S = 1.975 and N = 175.72) with a sensitivity (Δ*I*/Δ*C*) of 36.42 μA nM^−1^.

**Fig. 7:**
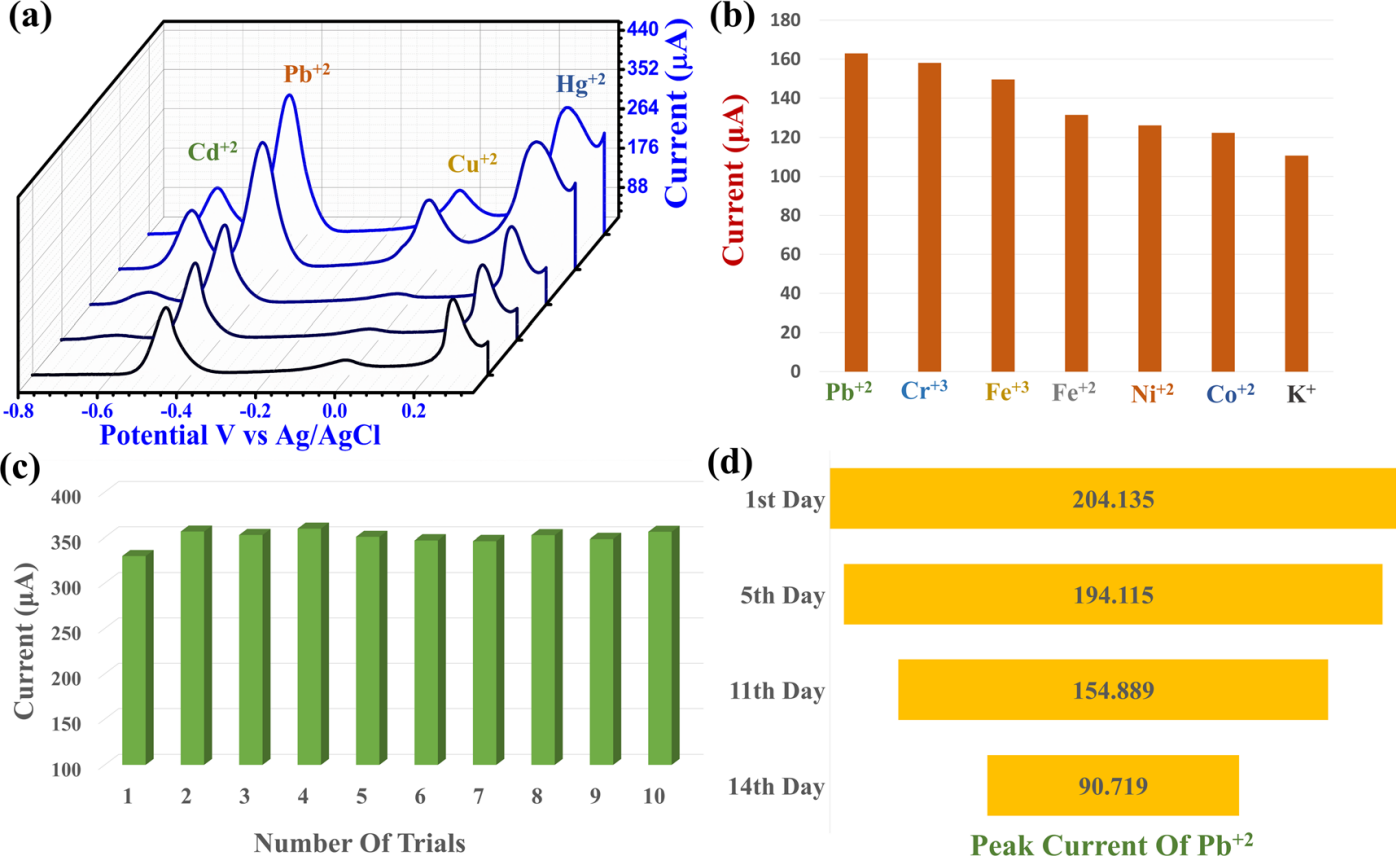
(a) SWASV for simultaneous detection of Cd^2+^, Pb^2+^, Cu^2+^ and Hg^2+^ under optimized SWASV conditions (100 to 350 nM). (b) Interference study of PPy/MoS_2_/GCE sensor with addition of equal concentrations (100 nM) of Pb^2+^, Cr^3+^, Fe^2+^, Fe^3+^, Ni^2+^, Co^2+^ and K^+^ for each trial. (c) SWASV reproducibility of the PPy/MoS_2_/GCE sensor under self-storage conditions. (d) Stability of the PPy/MoS_2_/GCE sensor for SWASV detection of 300 nM of Pb^2+^ in 0.1 M Na–Ac buffer solution, pH = 5.0, deposition potential = −0.9 V and deposition time = 9 min.

Simultaneous detection of different concentrations of Cd^2+^, Pb^2+^, Cu^2+^ and Hg^2+^ was carried out using the optimal parameters. Fig. 7a illustrates the PPy/MoS_2_/GCE response from 100 to 400 nM of Cd^2+^, Pb^2+^, Cu^2+^ and Hg^2+^ by SWASV. The positive or negative shift in the potential response at different concentrations of HMIs may be attributed to the competitive deposition between the HMIs during simultaneous detection. The stripping peaks for Cd^2+^, Pb^2+^, Cu^2+^ and Hg^2+^ appeared at −0.617, −0.446, −0.019 and 0.255 V *vs.* Ag/AgCl, respectively. The difference between the Cd^2+^ and Pb^2+^ peak currents was observed to be −0.173 V, between Pb^2+^ and Cu^2+^ it was −0.424 V and between Cu^2+^ and Hg^2+^ it was found to be 0.274 V *vs.* Ag/AgCl. The peak separation between the HMIs during this simultaneous detection indicates that PPy/MoS_2_/GCE is a potential electrochemical sensing catalyst for the simultaneous detection of HMI in drinking water.

To further evaluate the interference of possible metal ions on the SWASV response to Pb^2+^ a 100 nM concentration of Pb^2+^ and equal concentrations of Cr^3+^, Fe^2+^, Fe^3+^, Ni^2+^, Co^2+^ and K^+^ were tested each per trial (Fig. 7b). The relative standard deviation of Pb^2+^ was calculated as 14.324%. The reproducibility of the PPy/MoS_2_/GCE sensor was evaluated for ten consistent SWASV readings under a constant concentration of Pb^2+^ (300 nM) (Fig. 7c). The relative standard deviation of Pb^2+^ was calculated to be 2.30%, indicating the very reliable reproducibility of the fabricated sensor. The stability of the PPy/MoS_2_ was estimated under the self-storage environment. Fig. 7d shows the decrease in the peak current with day-wise stability. After 14 days, the peak current response was evaluated as 44.44%, which might be due to the deposition of a metal ion layer at the electrode surface or the exfoliation of the catalyst.

The above results indicate that functionalized PPy/MoS_2_ possesses excellent stability, reproducibility, simultaneous and individual detection of HMIs. A comparison of other reported catalysts and the PPy/MoS_2_ catalyst is given in Table 1. This shows that the developed PPy/MoS_2_ displays a much better/comparable performance for the detection of Pb^2+^.

**Table 1:** Comparison of PPy/MoS_2_ sensor with reported electrochemical catalysts for the detection of Pb^2+^

Sl. no.	Catalyst	Method of detection	LOD	Linear range	Ref.
1	Gr/PANI/SPE	SWASV	0.1 μg L^−1^	1–300 μg L^−1^	45
2	Pa/PPy/GO	DPV	0.4 μg L^−1^	5–150 μg L^−1^	46
3	F_3_O_4_ @ PANI	DPV	0.03 nM L^−1^	0.1 to 10^4^ nM L^−1^	47
4	G/PANI/PS	SWV	3.30 μg L^−1^	10–550 μg L^−1^	48
5	PANI-MC	ASV	4 nM L^−1^	20–1000 nM L^−1^	49
6	PANI-GO	FAAS	0.04 μg L^−1^	0.5–10 μg L^−1^	16
7	G/PANI	SWASV	0.1 μg L^−1^	1–300 μg L^−1^	15
8	PANA/CPE	DPV	7.12 × 10^−14^ M	1 × 10^−6^ to 10 × 10^−10^ M	50
9	Ag-PANI	SWV	0.04 μM	0.1–0.2 μM	17
10	PEDOT/NT	CV	2.33 μg L^−1^	5–100 μg L^−1^	51
11	PEDOT/Sb	SWV	1.8 μg L^−1^	—	52
12	rGO@CNT@Fe_2_O_3_/PPy	ASV	0.1 nM	0.02 to 0.26 μM	53
13	PA/PPy/ZIF-8@ZIF-67	ASV	2.9 nM	0.02 to 200 μM	
14	PPy-rGO	SWASV	4.7 × 10^−11^ M L^−1^	5 × 10^−9^ to 7.5 × 10^−7^ M L^−1^	54
15	PPy/UIO-66-NH_2_/GCE	DPASV	0.05 μg L^−1^	0.5 to 10 μg L^−1^	55
16	NiCo_2_O_4_@PPy/3D graphene	SWASV	0.2 nM	0.0125–0.709 μM	56
17	PA-doped PPy/MoS_2_	DPASV	1.78 μg L^−1^	10 to 300 μg L^−1^	57
18	PPy/Fe_3_O_4_ NBs	CV	0.4715 μM L^−1^	10 to 50 nM L^−1^	58
19	rGO/MWCNT/AuNP	DPV	7.1 × 10^−6^ nM m L^−1^	5 × 10^−5^–0.2 nM m L^−1^	59
20	Graphene sensors	ASV	0.5 μg L^−1^	30–100 μg L^−1^	60
21	Sn/SnO_2_	SWASV	2.15 μg L^−1^	6.2 and 20.7 μg L^−1^	61
22	Bi/GDY	DPASV	0.146 nM	10.0 nM to 100.0 μM	62
23	CoCu-MOF/PANI	ASV	0.39 μg L^−1^	—	63
24	MoS_2_/rGO-GCE	SWASV	0.005 μM	0.05 μM to 0.8 μM	64
25	2D-MoS_2_	SWASV	0.3 ppb	0 to 20 ppb	65
26	AuNPs/MoS_2_/GN	CV	1.0 μM	5.0 μM to 5.0 mM	66
27	CNT post-electrode	SWASV	2 nM	9.64 nM to 168.7 nM	67
28	Fe_3_O_4_@SiO_2_@IIP	DPV	0.05 ng mL^−1^	0.1 to 80 ng mL^−1^	68
29	Copper-based sensor	ASV	21 nM	10 μM to 25 nM	69
30	PEDOT:PSS/rGO	DPASV	0.09 ppb	1 ppb to 70 ppb	70
31	RGO–MNP	SWV	8.13 × 10^−10^ M	1.0 × 10^−9^ to 1.0 × 10^−3^ M	71
32	PPy/MoS_2_	SWASV	0.03 nM	0.02 to 300 nM	This work

## 4. Conclusion

In this work, an efficient electrochemical sensor based on the functionalization of CPs was fabricated for SWASV detection of HMIs. The nano-sphered PPy-functionalized MoS_2_ showed excellent sensitivity and selectivity. The developed electrochemical system was effectively analysed for individual and simultaneous detection of Pb^2+^ at trace levels using SWASV. Under optimized conditions, the PPy/MoS_2_/GCE system detected Pb^2+^ in the linear range of 0.02 to 300 nM, with an LOD of 0.03 nM and a sensitivity of 36.42 μA nM^−1^. The stripping peaks for Cd^2+^, Pb^2+^, Cu^2+^ and Hg^2+^ appeared at −0.617, −0.446, −0.019 and 0.255 V *vs.* Ag/AgCl, respectively. Furthermore, an anti-interference study with additional metal ions, such as Cr^3+^, Fe^2+^, Fe^3+^, Ni^2+^, Co^2+^ and K^+^, was studied and calculated to be 14.324% (RDS) with 44.44% retention in stability after 14 days. We believe that the functionalization of PPy with MoS_2_ may offer a potential candidate for the detection and analysis of heavy metals in drinking water.

## Data availability

The data supporting the findings of this study are available within the article files. Additional data can be accessed upon request from the corresponding author.

## Conflicts of interest

There is no conflict of interest to declare.

## Supplementary Material

RA-015-D4RA05688D-s001
