# Supplementary figures and images for: Polypyrrole functionalized MoS2 for sensitive and simultaneous determination of heavy metal ions in water
Source: RSC Adv. 2025 Jan 3;15(1):467–76. doi: 10.1039/d4ra05688d (PMC11697259; doi:10.1039/d4ra05688d)

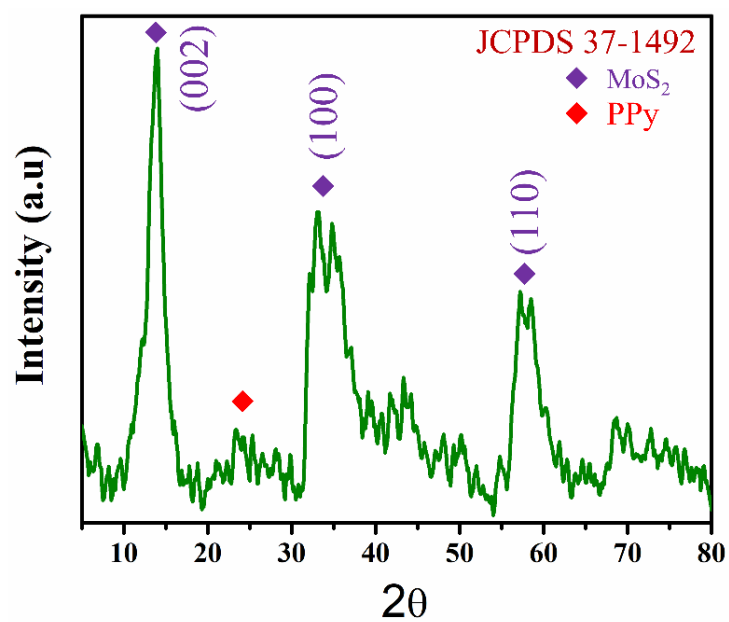

**Fig. 1** XRD of PPy/MoS<sub>2</sub>

Supplement: RA-015-D4RA05688D-s001 [file RA-015-D4RA05688D-s001.pdf]
